# Development of an allele-specific, loop-mediated, isothermal amplification method (AS-LAMP) to detect the L1014F *kdr-w* mutation in *Anopheles gambiae s. l.*

**DOI:** 10.1186/1475-2875-11-227

**Published:** 2012-07-06

**Authors:** Athanase Badolo, Kyioshi Okado, Wamdaogo M Guelbeogo, Hiroka Aonuma, Hironori Bando, Shinya Fukumoto, N’Fale Sagnon, Hirotaka Kanuka

**Affiliations:** 1 National Research Center for Protozoan Diseases, Obihiro University of Agriculture and Veterinary Medicine, Inada-cho, Obihiro, Hokkaido, 080-8555, Japan; 2 University of Ouagadougou, BP 7021, Ouagadougou 03, Burkina Faso; 3 Centre National de Recherche et de Formation sur le Paludisme, BP 2208, Ouagadougou 01, Burkina Faso; 4 Department of Tropical Medicine, The Jikei University School of Medicine, 3-25-8, Nishi-Shinbashi, Minato-ku, Tokyo, 105-8461, Japan

**Keywords:** Insecticide, Resistance, *kdr*, LAMP, Malaria, Mosquito

## Abstract

**Background:**

Malaria control relies heavily on treated bed nets and indoor residual spraying with pyrethroid insecticides. Unfortunately, the resistance to pyrethroid insecticides, mainly due to the *kdr* mutation, is spreading in the main malaria vector *Anopheles gambiae s.l.,* decreasing the insecticides’ efficacy. To manage the insecticide resistance rapidly and flexibly, simple and effective tools for the early detection of resistant mosquitoes are needed. This study aimed to develop an allele-specific, loop-mediated, isothermal amplification (AS-LAMP) method to detect the West African-type *kdr* mutation (*kdr-w*; L1014F) in field-collected mosquitoes.

**Methods:**

DNA fragments of the wild-type and the mutated *kdr* gene were used to select the primers and develop the method. The primers were designed with the mutation at the 5’ end of the backward inner primer (BIP). The AS-LAMP method was compared to the AS-PCR method using the genomic DNA of 120 field-collected mosquitoes.

**Results:**

The AS-LAMP method could discriminate between the wild-type homozygote, the heterozygote, and the *kdr-w* homozygote within 75 min. The AS-LAMP method has the advantage of being faster and at least as sensitive and specific as the AS-PCR method.

**Conclusions:**

The AS-LAMP method can be used to detect the *kdr* mutation for quick decision-making, even in less well-equipped laboratories.

## Background

The prevention of malaria has relied mainly on controlling its vectors by using pyrethroid insecticides for both bed nets and indoor sprays. Considerable effort is being made to reach the goal of 80% insecticide-treated net (ITN ) coverage by 2015[1], and the number of African children protected by ITNs in a stable malaria-endemic area increased from 1.7 million (1.8%) in 2000 to 20.3 million (18.5%) in 2007 [2]. However, continued progress in deploying these malaria-controlling tools is threatened by vector resistance against the insecticides, which could compromise the efficacy of the treated bed nets and indoor residual insecticide spray across Africa [3-9]. In fact, recent studies have demonstrated that mosquito resistance to pyrethroid insecticides in Benin [10] and in Equatorial Guinea [11] has decreased the efficacy of ITNs and indoor residual sprays. In addition, Verhaeghen et al [12] have demonstrated that *kdr* resistance mutation has an epidemiological impact with higher frequency of this mutation in *Plasmodium falciparum*-infected mosquito than the non-infected.

The *kdr* mutation is the primary cause of resistance to pyrethroids and DDT among *Anopheles gambiae s.l.* and many other insects [9,13-15]. Martinez-Torres *et al*[15] showed that this resistance in western Africa was due to a single nucleotide change (A to T) in the gene encoding the voltage-dependent sodium channel. This mutation results in a leucine (TTA) to phenylalanine (TTT) change at position 1014 (L1014F). This so-called West African-type *kdr* mutation (*kdr-w*) is widespread in western and central Africa, with a gene frequency close to 92% in the S molecular form of *An. gambiae*[16,17].

Several methods have been developed to detect the *kdr* mutation in field-collected mosquitoes, including an allele-specific (AS)-PCR method [9,15], which is the most widely used. Other methods include the heated oligonucleotide ligation assay (HOLA), sequence-specific oligonucleotide probe (SSOP)-ELISA, PCR-Dot Blot, fluorescence resonance energy transfer FRET/Melt Curve analysis, and PCR elongation with fluorescence, but all these methods require heavy and/or expensive instruments, such as a thermal cycler [18]. The sensitive, specific, and rapid detection of mutations associated with insecticide resistance is prerequisite for resistance management, in that this information allows national vector control units to adapt their strategies according to the resistance level.

Loop-mediated isothermal amplification (LAMP), a DNA amplification method, was developed within the last 10 years [19] as an alternative to conventional PCR. Compared with conventional PCR methods, LAMP is more sensitive, specific, rapid, and cost-effective [20,21]. LAMP uses four different primers - two inner primers (FIP and BIP), and two outer primers (F3 and B3) - that are specifically designed to recognize six distinct regions on the target DNA, thereby increasing both the sensitivity and specificity of the detection [19,22]. The reaction proceeds at a constant temperature using the strand-displacement property of the *Bst* DNA polymerase, the only enzyme used in the test. LAMP has been successfully used to detect *Plasmodium berghei* in *Anopheles stephensi*[23], and *Dirofilaria immitis* and Flock House Virus in *Aedes aegypti*[24,25] It was recently used to identify the two species of the *An. gambiae* complex [26]. This method has also been successfully used to type single nucleotide polymorphisms in a DNA sequence [20,27]. The development of an allele-specific (AS)-LAMP method to distinguish the two types of *kdr* genes (West African and wild type) could be very useful for malaria vector control in less developed countries, because LAMP is faster than conventional PCR, and can be performed with minimal equipment. In the present study, a LAMP-based allele distinction method is developed to identify the West African-type *kdr* mutation responsible for the resistance to pyrethroid insecticides in field-collected mosquitoes.

## Methods

### Primer design for AS-LAMP

As a model for designing the primers, multiple alignments from published sequences of the sodium channel gene were used [15,28]. Two sets of LAMP primers were designed manually to distinguish the two different nucleotides in the gene sequence for position 1014 of the sodium channel (L1014F). Two BIP primers were designed as specific primers, with the mutation on the 3’ end of the B2 primer (bold type) (5’ end of the BIP primer) and an additional mismatched nucleotide (lower-case letter) to increase the specificity to each targeted nucleotide site. The other three primers, F3, B3, and FIP, were the same for the two primers sets. The sequences of the primers were as follows: F3, ATG ATC TGC CAA GAT GGA AT**,** B3: AAA CGA TCT TGG TCC ATG T; FIP (F1c-F2), ATC CCA CAT TGA TTC AAT C-GC ATT CCT TCA TGA TTG TGT TCC; BIP-wild (B1c-B2), TGC TTG TCG GTG ATG TAT CCT GC-T AAT TTG CAT TAC TTA CGA g**T**; BIP-*kdr-w* (B1c-B2), TGC TTG TCG GTG ATG TAT CCT GC-T AAT TTG CAT TAC TTA CGA g**A**.

### Preparation of plasmid DNA as an AS-LAMP template

DNA fragments from *An. gambiae* mosquitoes carrying either the wild-type or West Africa-type *kdr* gene were used. The target sequence was amplified using PCR primers (FP (KO80): GAT AGA TTC CCC CAC CAT GA and BP (KO81): CTC ATT ATC TGC CGT TGG TG), the PCR products were separated by gel electrophoresis, and the products were extracted using the QIAEX kit (Qiagen). The amplified DNA fragments were inserted into the *Eco*RV site of the pBSSK vector and introduced into *Escherichia coli* competent cells (DH5α) grown in SOC medium. The cells were pre-cultured, and the plasmid insertion was confirmed by cutting the plasmid using *Eco*RI. The transformed cells were then subjected to large-scale culture for plasmid DNA extraction and purification. These plasmids were purified using the Qiagen kit for plasmid purification and adjusted to 0.2 μg/μl for the stock. The insertion in these plasmids was confirmed using the restriction enzymes *Bst*XI and *Xho*I and by sequencing using the BSR (5’-TGT GGA ATT GTG AGC GGA TAA-3’) or BSF (5’-TTT TCC CAG TCA CGA CGT TG-3’) universal primers. The ABI Prism BigDye terminator cycle protocol was used for the sequencing. The sequences of these plasmids were aligned and compared to the gene sequence from the database using CLC Sequence Viewer 6.

### Extraction of DNA from field-collected mosquitoes

The genomic DNA of field-collected mosquitoes was extracted by homogenizing individual mosquito with a plastic homogenizer in 100 μl Buffer A (0.1 M Tris HCl (pH 9.0), 0.1 M EDTA, 1% SDS, and 0.5% DEPC (diethyl pyrocarbonate)) and incubating the homogenate for 30 min at 70 °C. After incubation, 22.4 μl of 5 M KoAc (potassium acetate) was added, and the mixture was cooled on ice for 30 min. After centrifugation at 20,000 x g for 15 min at 4 °C, the DNA-containing supernatant was transferred into a new tube and mixed with 45 μl isopropanol. The solution was centrifuged at 20,000 x g for 20 min at 4 °C, and the supernatant was discarded. The DNA pellet was rinsed with 70% ethanol and dried. The pellet was then diluted in 30 μl TE buffer.

### AS-LAMP detection of the *kdr-w* mutation

The AS-LAMP reaction was conducted following the manufacturer’s recommendations (Eiken Chemicals Co, Japan). A master mix was prepared using 6.25 μl of 2x reaction mix (2.8 mM each dNTP, 40 mM Tris–HCl (pH 8.8), 20 mM KCl, 16 mM MgSO_4_, 20 mM (NH_4_)_2_SO_4_, 0.2% Tween 20, and 1.6 M Betaine), 2.75 μl distilled water, 0.5 μl of each primer, and 0.5 μl of the *Bst* DNA polymerase (4 U). The concentration of the primers was 40 pmol/μl for the inner primers (BIP and FIP) and 5 pmol/μl for the outer primers (B3 and F3). To a test tube was added 11.5 μl of the master mix and either 1 μl of DNA solution or 1 μl of distilled water (negative control). All the procedures were performed on ice.

Each tube was then put into the Loopamp® real time turbidimeter (Eiken Chemicals Co, Japan) at 63 °C, and the turbidity was monitored. The reaction was terminated by heating the tube at 95 °C for five minutes. The LAMP products were subjected to 2% agarose gel electrophoresis at 100 V, and the gel was stained with ethidium bromide and examined under UV light to check the amplified fragments.

### AS-PCR detection of the *kdr-w* mutation

The allele-specific PCR (AS-PCR) protocol of the Malaria Research and Reference Reagent Resource Center (MR4) [29] was used to detect the *kdr-w* mutation. The *kdr* mutation region was amplified with four primers as previously reported [29]: IPCF (2.5 pmol/μl), CTA ACG CGA ATT AAA TGC TTT GTG ACAG; IPCR (2.5 pmol/μl), CAA AAG CAA GGC TAA GAA AAG GTT AAG C; WT (1 pmol/μl), GGT CCA TGT TAA TTT GCAT TAC TTA CGA aTA; West (8.8 pmol/μl), CTT GGC CAC TGT AGT GAT AGG AAA TgTT. The PCR reaction tube contained 0.5 μl *Taq* DNA polymerase (5 U/μl), 2.5 μl 10x PCR Buffer (containing 15 mM MgCl_2_), 2.5 μl dNTP (2–2.5 mM mix), 2.0 μl 25 mM MgCl_2_, and 1 μl of each primer. Amplification was performed with 1 μl genomic DNA as the template. The PCR programme was 95 °C/5 min x 1 cycle, (95 °C/30 sec, 61 °C/30 sec, 72 °C/30 sec) x 30 cycles, 72 °C/5 min x 1 cycle, and 4 °C hold. After the reaction, 5 μl of the PCR product was run on a 2% agarose gel and stained with ethidium bromide. Three types of mosquito genome (+/+, homozygous for wild type; *kdr-w*/*kdr-w*, homozygous for *kdr-w*; *kdr-w*/+, heterozygous for *kdr-w*) were identified using the AS-PCR and direct sequencing, and used as DNA templates for further experiments.

### Direct sequencing of mosquito DNA

DNAs from *An. gambiae* mosquitoes were used as PCR templates. The target sequence was amplified using the PCR primers KO80 and KO81 as described above. PCR was performed in a tube with *Ex Taq* polymerase (0.5 μl), 10x *Ex Taq* buffer (2.5 μl), 2.5 mM dNTP (2.5 μl), 0.5 μl of each primer, 17.5 μl = distilled water, and 1 μl genomic DNA (concentration 0.1 μg/μl). The PCR programme was 95 °C/5 min x 1 cycle, (95 °C/30 sec, 61 °C/30 sec, 72 °C/30 sec) x 30 cycles, 72 °C/5 min x 1 cycle, and 4 °C hold. The PCR product was purified using a YM100 Microcon® Millipore extraction column, and the final product was diluted in 30 μl of distilled water. The nucleotide sequences were determined with the BigDye terminator sequencing kit v3.1 (Applied Biosystems) using an automated 3100 genetic analyzer.

### Sensitivity and specificity of mutation detection

We considered the sequencing as the standard method for detecting the *kdr* mutation for true positives and true negatives for each mutation. The sensitivity is defined as the ratio of true positives to combined true and false positives and the specificity as the ratio of true negatives to combined true negative and false positive. The calculation of specificity and sensitivity confidence limits of AS-LAMP and AS-PCR have been produced with the Wilson score method [30].

## Results

### AS-LAMP detection of the *kdr-w* mutation in plasmid DNA

**Figure 1:**
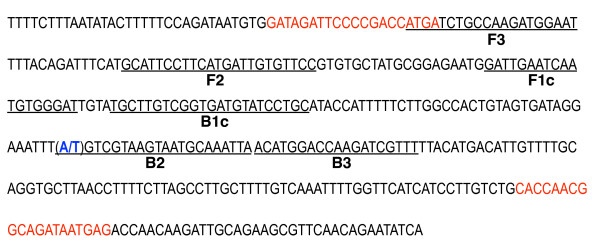
**DNA sequence of part of the voltage-dependent sodium channel gene surrounding the*kdr*mutation, and the position of the primers designed for AS-LAMP.** Partial *kdr* gene sequence and location of primers, FIP (F1c-F2), BIP (B1c-B2), F3, and B3. The nucleotides in red are the regions of the PCR primers used for the PCR product inserted into the plasmid. The mutated nucleotide in *kdr-w* is shown in bold blue type, A for the wild type and T for the West African-type (*kdr-w*).

**Figure 2:**
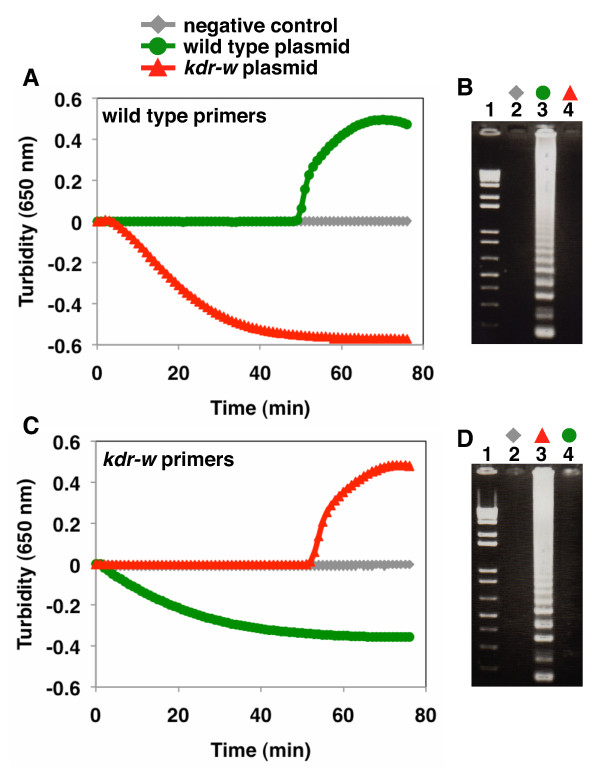
**AS-LAMP detection of the*kdr-w*mutation in plasmid DNA.** (**A**) Amplification of the target sequence (plasmid) with the wild-type primer set monitored by a real-time turbidimeter (turbidity at 650 nm); wild-type plasmid (green), *kdr-w* plasmid (red), and negative control (grey). (**B**) Agarose-gel electrophoresis of the AS-LAMP-amplified products from (A). Lane 1, DNA markers; 2, negative control; 3, wild-type plasmid; 4, *kdr-w* plasmid. (**C**) Amplification of the target sequence (plasmid) with the *kdr-w* primer set monitored by a real-time turbidimeter (turbidity at 650 nm); wild-type plasmid (green), *kdr-w* plasmid (red), and negative control (grey). (**D**) Agarose-gel electrophoresis of the AS-LAMP-amplified products from (C). Lane 1, DNA markers; 2, negative control; 3, *kdr-w* plasmid; 4, wild-type plasmid.

To detect the single nucleotide difference in *kdr-w* by AS-LAMP, the BIP primers were designed to bind the specific SNP with an additional mismatched nucleotide on the penultimate nucleotide of the BIP 3’ end [Figure 1 and see Methods]. The mismatched nucleotide increases the specificity of the BIP primer for its target DNA. The primers designed for AS-LAMP appropriately distinguished the wild-type from the West African-type mutation when plasmid *kdr* DNA was tested [Figure 2]. The detection time for the wild-type *kdr* gene was around 50 min after incubation was started in the turbidimeter, using the wild-type primers. No amplification of *kdr-w* was seen when the wild-type primers were used, even after 75 min of incubation [Figure 2, A]. For the *kdr-w* detection, amplification was detectable around 52 min after the incubation was started, and these primers did not amplify the wild-type sequence at all, even after 75 min [Figure 2, C]. Gel electrophoresis of the LAMP products confirmed that the primers designed for the detection of each *kdr* gene amplified the appropriate DNA fragments [Figure 2, B and D].

### AS-LAMP detection of the *kdr-w* mutation in mosquito genomic DNA

**Figure 3:**
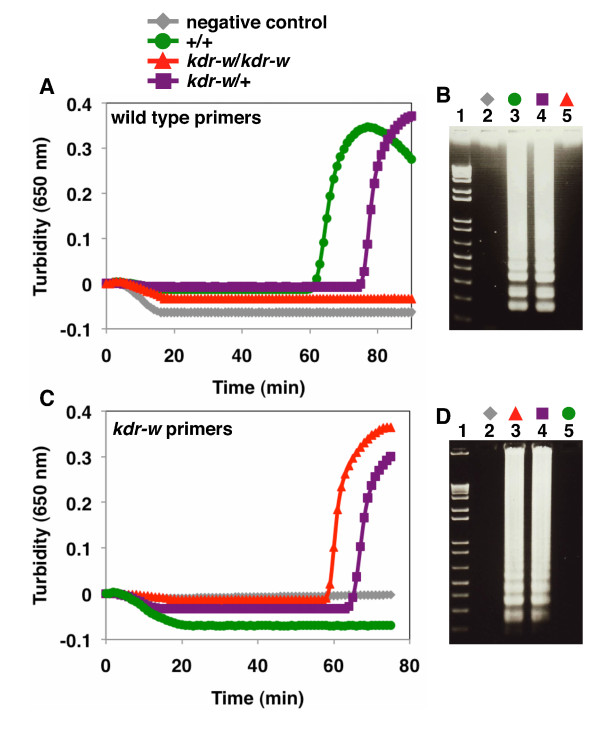
**AS-LAMP detection of the*kdr-w*mutation in mosquito genomic DNA.** (**A**) Amplification of the target sequence (mosquito genomic DNA) with the wild-type primer set monitored by a real-time turbidimeter (turbidity at 650 nm); +/+ (green), *kdr-w/+* (violet), *kdr-w/kdr-w* (red), and negative control (grey). (**B**) Agarose-gel electrophoresis of the AS-LAMP-amplified products from (A). Lane 1, DNA markers; 2, negative control; 3, +/+; 4, *kdr-w/+*; 5, *kdr-w/kdr-w*. (**C**) Amplification of the target sequence (mosquito genomic DNA) with the *kdr-w* primer set monitored by a real-time turbidimeter (turbidity at 650 nm); +/+ (green), *kdr-w/+* (violet), *kdr-w/kdr-w* (red), and negative control (grey). (**D**) Agarose-gel electrophoresis of the AS-LAMP-amplified products from (C). Lane 1, DNA markers; 2, negative control; 3, *kdr-w/kdr-w*; 4, *kdr-w/+*; 5, +/+.

The genomic DNA extracted from *An. gambiae* was used and the results were comparable to those obtained when plasmid DNA was used as the template [Figure 3]. In addition, the primers were able to discriminate three patterns of mosquito genotypes: the two homozygous genotypes (+/+ and *kdr-w*/*kdr-w*) and heterozygous genotype (*kdr-w/+*). Using the wild-type primers, the detection times were 60 min for the wild-type homozygote (+/+) and 75 min for the heterozygote (*kdr-w/+*) [Figure 3, A], and neither the negative control nor the *kdr-w* homozygotic (*kdr-w*/*kdr-w*) sequences were amplified [Figure 3, A]. The detection time using the *kdr-w* primers was 60 min for the *kdr-w* homozygote (*kdr-w*/*kdr-w*), and 65 min for the heterozygote (*kdr-w/+*) [Figure 3, C], and no amplification was observed for either the negative control or the wild-type homozygote (+/+) [Figure 3, C]. The gel electrophoresis of the LAMP products confirmed the results of the observed turbidity [Figure 3, B and D].

### Species, molecular forms, and *kdr* type identification in field-collected mosquitoes

The AS-LAMP method was next applied to assess the genotype of *Anopheles* mosquito with respect to the *kdr-w* mutation in samples collected from Burkina Faso. A total of 120 mosquitoes were tested using RLFP-PCR [31] to identify the *An. gambiae* species and the molecular form of *An. gambiae s.s*. Of these mosquitoes, 47% were identified as *Anopheles arabiensis* and 53% as *An. gambiae s.s.,* of which 15% were of the S molecular form and 85% of the M molecular form.

When AS-PCR was performed to detect the *kdr-w* mutation in the 120 field-collected mosquito samples, the results showed that 73 were homozygous for wild type (+/+), 18 heterozygous for *kdr-w* (*kdr-w/+*), 20 homozygous for *kdr-w* (*kdr-w/ kdr-w*), and nine unidentified [Table 1]. The sodium channel surrounding *kdr* region in these samples was sequenced [Table 1]. The samples were then subjected to the AS-LAMP analysis to detect the *kdr* mutation.

**Table 1:** **Sensitivity and specificity of the AS-PCR and AS-LAMP methods for*kdr*mutation detection**

Genotype^*^	Detection Method			Sensitivity (95% CL)		Specificity (95% CL)	
	Sequencing	AS-PCR	AS-LAMP	AS-PCR	AS-LAMP	AS-PCR	AS-LAMP
*kdr*-w/*kdr*-w	20	20	23	0.83 (0.64 - 0.93)	0.92 (0.74 - 0.98)	1 (0.96 - 1)	0.99 (0.94 - 1)
*kdr*-w/*+*	26	18	20	0.94 (0.73 - 0.99)	0.94 (0.73 - 0.99)	0.98 (0.92 - 0.98)	0.96 (0.90 - 0.98)
*+*/*+*	70	73	70	0.93 (0.85 - 0.97)	0.99 (0.92 - 1)	0.94 (0.83 - 0.98)	1 (0.92 - 1)
Unidentified	4	9	7	-	-	-	-
Total	120	120	120	-			

Genotypes were analysed by sequencing, AS-LAMP, and AS-PCR. The sensitivity and specificity of the AS-PCR and AS-LAMP methods relative to sequencing are expressed as proportions, and their 9~~confidence limits (CL) are shown in parenthesis.

The sensitivity of each method (AS-LAMP and AS-PCR) for detecting the *kdr* genotypes compared to sequencing was calculated [Table 1]. AS-LAMP and AS-PCR showed high sensitivity and specificity for detecting the wild-type homozygous (+/+) and the heterozygous mosquitoes (*kdr-w/+*). The sensitivity of these two methods was similar for detecting the heterozygote (0.94, confidence limits: 0.73-0.99). The AS-LAMP method was more sensitive than AS-PCR for detecting the homozygotes (0.92 and 0.83 for *kdr-w/kdr-w*; 0.99 and 0.93 for the +/+, respectively) but the difference between these two methods was not statistically significant. In addition, the AS-LAMP method had fewer non-detectable samples than AS-PCR (0.058 and 0.075, respectively). The specificity of the two methods was comparable for detecting the *kdr-w* homozygote (*kdr-w*/*kdr-w*) (1.0 and 0.99 for AS-PCR and AS-LAMP, respectively). For the heterozygote (*kdr-w/+*), the detection specificity was 0.98 and 0.96 for AS-PCR and AS-LAMP, respectively. The AS-LAMP method showed better specificity than the AS-PCR for identifying the wild-type homozygote (1.0 and 0.94, respectively), but this difference was not statistically significant. These data indicated that the AS-LAMP method developed in this study was an appropriate alternative method for detecting the insecticide resistance of mosquitoes at the genetic level.

## Discussion

The resistance developed to insecticides is affected by local conditions, like cotton cultivation or vegetable gardening [17,32]. Even in the absence of insecticide pressure, the level of resistance can vary from one area to another, and differences result from the distinct sensitivities of vectors to different families of insecticides [8]. The future of malaria vector control relies not only on new insecticides but also on managing the resistance to insecticides that are suitable for a particular area. A sensitive and specific method that requires minimal specialized equipment is prerequisite for resistance management at the local level.

In the present study, an allele-specific LAMP method was developed to target the *kdr-w* mutation, which is responsible for the resistance of mosquitoes to pyrethroid insecticides in west and central Africa [3,7,33]. The specific primers designed for the mutation and an additional mismatched nucleotide at the 3’ end of the BIP primer allowed the specific amplification of either the wild-type or the West African-type *kdr* gene, using plasmid DNA as the template. More importantly, even when mosquito genomic DNA was used, the primers successfully distinguished the *kdr-w* homozygote from the heterozygote in less than 90 min. Since general LAMP method requires only a water bath to amplify DNA, and the result is detectable by the naked eye, the AS-LAMP method could be applied in less well-equipped laboratories to facilitate insecticide selection for bed net treatment or indoor residual insecticide spraying. Vector resistance to insecticide should be considered a constant problem, and monitored regularly. The AS-LAMP method, which was at least as sensitive and specific as AS-PCR, can be used for this purpose; that is, this method can be used in situations where conventional PCR is difficult. The spread of another *kdr* mutation (East African-type, *kdr-e* (L1014S)) in malaria-endemic areas reveals the need to develop a multiplex AS-LAMP method that targets the *kdr-e* mutation as well as *kdr-w* one.

Poon *et al*[34] and Bonizzoni *et al*[26] both reported that LAMP is more cost-effective than conventional PCR. For detecting the *kdr* mutation, the AS-LAMP was faster than the conventional AS-PCR method: LAMP took only 75 min, compared to more than three hours for the PCR. In addition, the LAMP method avoids the need for ethidium bromide, a hazardous chemical used for staining DNA.

## Conclusions

An AS-LAMP method was developed to detect the West African-type *kdr* mutation in mosquitoes. This method can be used to rapidly detect the *kdr* mutation, even in less well-equipped laboratories.

## Competing interests

The authors declare that they have no competing interests.

## Authors' contributions

AB and HK conceived the study and wrote the paper. AB designed the primers and carried out the molecular study. OK, HA and SF performed experiments in the laboratory. AB, NS, WMG, OK and HB contributed to the fieldwork related to collecting the *Anopheles* mosquitoes. All the authors read and approved the final manuscript.
